# Healthcare Information Management and Accreditation in Europe

**DOI:** 10.3390/healthcare14060748

**Published:** 2026-03-16

**Authors:** Radu Ilinca, Laura Iosif, Dan Adrian Luțescu, Mircea Valentin Trică, Ionela Ganea, Tudor-Claudiu Spînu, Ana-Maria Cristina Țâncu

**Affiliations:** 1 Department of Medical Informatics and Biostatistics, Faculty of Dentistry, “Carol Davila” University of Medicine and Pharmacy, 4–6 Eforie St., 050037 Bucharest, Romania; radu.ilinca@umfcd.ro (R.I.); mircea-valentin.trica@drd.umfcd.ro (M.V.T.); 2 Department of Prosthodontics, Faculty of Dentistry, “Carol Davila” University of Medicine and Pharmacy, 37 Dionisie Lupu Street, District 2, 020021 Bucharest, Romania; laura.iosif@umfcd.ro (L.I.); tudor.spinu@umfcd.ro (T.-C.S.); anamaria.tancu@umfcd.ro (A.-M.C.Ț.); 3 3rd Modern Languages Department, Faculty of Medicine, “Carol Davila” University of Medicine and Pharmacy, 37 Dionisie Lupu Street, District 2, 020021 Bucharest, Romania

**Keywords:** healthcare information governance, diagnostic service management, health policy implementation, governance, reimbursement, accreditation

## Abstract

**Highlights:**

**What are the main findings?**

- National healthcare systems operate within a harmonized European accreditation framework, while national rules specify how accredited paraclinical healthcare information is referenced in routine clinical and administrative documentation
- The differences in communication influence how clinicians, healthcare managers, and payers identify the recognition, usability, and reimbursement status of diagnostic information.

**What are the implications of the main findings?**

- Accreditation-related communication functions as a governance and management instrument by shaping healthcare financing decisions, operational workflows, and policy implementation.
- The improvement of the transparency and consistency in the communication of accredited status may enhance healthcare efficiency, support informed decision-making, and may reduce ambiguity in cross-sector healthcare interactions.

**Abstract:**

Background/Objectives: Healthcare systems increasingly rely on standardized diagnostic information to support clinical decision-making, reimbursement, and public health governance. Although accreditation of medical laboratories underpins trust in diagnostic services, in practice, it is encountered primarily through the way in which accredited status is communicated in routine healthcare documentation. This study examines national rules that govern the communication of accreditation-related information and their relevance for healthcare management and policy. Methods: A descriptive, document-based comparative analysis was conducted across all 42 national accreditation bodies participating in the European Co-operation for Accreditation Multilateral Agreement (EA-MLA). Official regulations and policies governing the use of accreditation symbols and references in medical laboratory documentation were analyzed. Only documents confirmed as valid and in force as of January 2026 were included. The analysis focused on report-level identification, differentiation of accredited and non-accredited results, use beyond reports, and consequences of misuse. Results: Across countries, accreditation communication rules define how laboratory results are recognized, reimbursed, and operationally used within healthcare systems. While regulatory detail varies, common requirements exist regarding clear identification of accredited results and safeguards against misinterpretation, which directly influence administrative processes and financing mechanisms. For example, in some healthcare systems, accredited reporting determines eligibility for public reimbursement, while in others, it constitutes a legal prerequisite for providing laboratory services. Conclusions: Accreditation-related communication functions as an element of healthcare information governance rather than a purely technical marker. National variations reflect healthcare policy and management priorities, with implications for efficiency, transparency, and access to care.

## 1. Introduction

Modern healthcare systems operate under sustained pressure to deliver safe, effective, and equitable care while managing constrained financial and human resources. Clinical decision-making, service planning, reimbursement mechanisms, and health policy implementation increasingly depend on standardized information generated by healthcare providers, particularly by diagnostic and laboratory services. In this context, the credibility and interpretability of healthcare documentation are not merely technical concerns but integral elements of healthcare governance, financing, and patient trust [1,2,3,4,5].

Like many complex services, healthcare users—patients, clinicians, healthcare managers, and payers—generally cannot directly evaluate technical quality before a service is provided and often, not even after receiving results. Instead, they rely on indirect indicators in healthcare documentation to determine whether predefined requirements have been met [6,7,8,9]. These indicators may appear as standardized wording, symbols, or formal references issued by independent third parties [10,11,12,13]. Their goal is not to explain how quality was achieved, but to indicate that specific conditions have been satisfied; therefore, allowing users to rely on this information for clinical, managerial, or financial decisions in environments marked by uncertainty and information asymmetry [13,14].

Laboratory reports serve as a key and impactful interface between highly specialized technical procedures and their users. While maintaining analytical accuracy is crucial, the way in which laboratory data is presented—the way in which results are labeled, contextualized, and qualified—significantly affects interpretation and its subsequent use. This is particularly relevant in healthcare systems, where laboratory results can lead to further actions, such as treatment decisions, eligibility for reimbursement, insurance coverage, or inclusion in public health surveillance and screening programs [14,15,16]. In these environments, documentation practices become an essential part of healthcare operations rather than merely administrative formalities.

From a healthcare perspective, accreditation can be seen as a structured process through which independent verification fosters trust in healthcare services. For medical laboratories, this verification typically follows ISO 15189 [17], an international standard outlining requirements for quality management, technical competence, and patient-centered laboratory services [16]. However, for most healthcare professionals and patients, accreditation is not perceived as an assessment process, but as a communication signal integrated into routine documentation. This is exemplified by the widely used principle “accredited once, accepted everywhere,” which supports international and regional recognition systems for accredited healthcare services [18,19]. This frequently cited maxim, “accredited once, accepted everywhere,” should be understood as an administrative and governance principle rather than a statement of clinical interchangeability. Within the European accreditation infrastructure, this principle refers to the mutual recognition of accreditation decisions issued by national accreditation bodies under multilateral arrangements; therefore, enabling accredited results to be formally accepted as a matter of administrative recognition for defined regulatory, administrative, and reimbursement purposes, without requiring repeated accreditation-related verification. Notably, this recognition does not imply uniform clinical interpretation or equivalence of diagnostic outcomes across healthcare settings. Clinical decision-making remains dependent on local clinical guidelines, patient context, and professional judgment. In practice, the maxim operates primarily at the level of information acceptance and usability, such as eligibility for reimbursement, inclusion in care pathways, or acceptance in cross-border administrative processes, rather than a guarantee of identical clinical meaning or application. Across healthcare systems, the practical consequences associated with accreditation-related communication vary, ranging from eligibility conditions to formal requirements or more neutral administrative roles, as examined in the comparative analysis presented in this study.

References to accredited status inform users—such as referring physicians, healthcare providers, managers, and payers—that results were produced under recognized conditions and are suitable for specific uses. In many healthcare systems, this distinction carries concrete operational and financial consequences. In Romania, for example, only laboratory results issued under accredited conditions are eligible for reimbursement by the national health insurance system [20]. In other systems, accreditation is not merely linked to additional benefits, but constitutes a prerequisite for providing the service itself. In France, medical laboratories are legally required to operate under ISO 15189 accreditation; therefore, they make accreditation a baseline condition for service provision rather than an optional quality enhancement [21]. In such contexts, accreditation-related communication directly affects access to healthcare services, patient pathways, and the flow of healthcare financing.

Healthcare systems are increasingly interconnected, shaped by patient mobility, cross-border care, shared clinical guidelines, and international public health challenges. While institutional frameworks exist to facilitate mutual recognition of accredited healthcare services across jurisdictions, the practical meaning of these frameworks depends on how accreditation-related information is communicated in routine healthcare documentation. National rules governing the use of symbols, wording, and references determine how clearly healthcare users can identify whether results are recognized, reimbursable, or subject to specific policy conditions [17,18,19].

Beyond laboratory medicine, similar governance challenges arise in other diagnostic domains where standardized documentation mediates clinical, organizational, and regulatory decision-making. In particular, radiological examinations are subject to European-level health and organizational surveillance following the transposition of Directive 2013/59/EURATOM, which has highlighted the importance of structured information flows, traceability, and organizational health literacy in radiological services. Recent work has emphasized how these documentation and governance mechanisms support effective implementation of radiation-protection frameworks within healthcare systems, by reinforcing the broader relevance of accreditation-related communication beyond laboratory testing [22].

**Figure 1:**
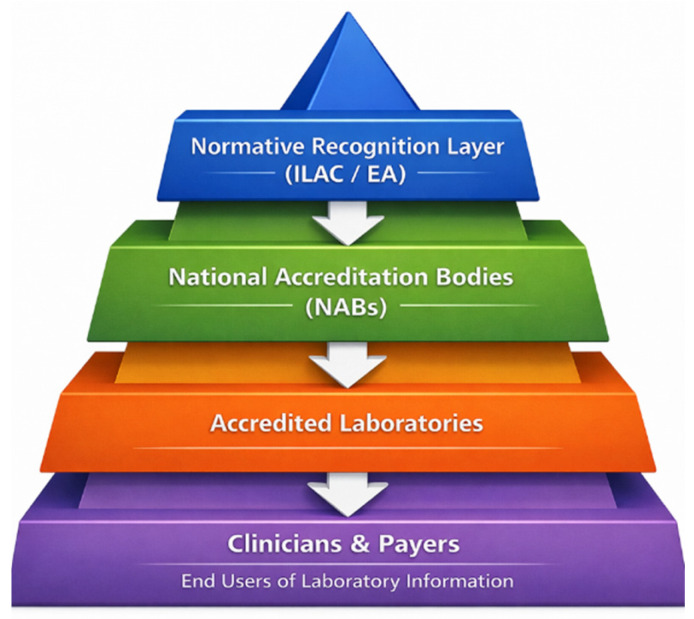
Accreditation governance pyramid.

Figure 1 schematically illustrates: (i) the governance structure through which accreditation operates in healthcare, from international and regional recognition frameworks (ILAC/EA), through national accreditation bodies, to accredited laboratories and (ii) the downstream users of healthcare information, including clinicians and payers.

Despite the importance of these communication practices, existing research has largely focused on accreditation as an instrument of quality assurance or organizational improvement [23,24,25,26]. Far less attention has been paid to the way in which accreditation is operationalized as a communication tool within healthcare systems, particularly how reporting practices vary across countries, how accredited and non-accredited information is distinguished, and how these distinctions intersect with healthcare management and financing [26,27,28,29]. These variations do not necessarily reflect differences in analytical quality, but they may influence the interpretation, the acceptance, and the use of healthcare information by clinicians, managers, and payers [29].

The present study addresses this gap by providing a descriptive and comparative analysis of national rules that govern the communication of accreditation-related information in healthcare laboratory documentation across multiple countries. Rather than assessing accreditation systems or their effectiveness, the analysis focuses on the way in which accreditation is communicated in practice: the way in which reporting rules define the use of symbols or references, the way in which mixed sets of results are presented, and under which conditions accreditation-related information may appear beyond formal laboratory reports. By situating accreditation within the broader context of healthcare communication and management, this study contributes to ongoing discussions on transparency, trust, and information governance in contemporary healthcare systems.

The objective of this review is to examine the way in which formal rules governing accredited status are communicated through laboratory test reports and related healthcare documentation, and how these communication practices shape operational excellence, healthcare management, and financing mechanisms within healthcare systems. The study does not aim to evaluate accreditation systems themselves or laboratories’ technical performance. The focus is exclusively on the communicative and governance dimensions of accreditation.

## 2. Materials and Methods

This review employed a comprehensive, descriptive, document-based methodology to analyze how accreditation-related information is communicated in healthcare laboratory documentation across countries that do participate in the European accreditation framework. The review comprises all 42 national regulations/policies/guidelines on the topic. It is worthwhile mentioning that all 27 European Union (EU) member states, 8 associated EU states, and 6 non-EU yet EA-MLA members are included in the analysis.

In Europe, accreditation is organized as a pyramidal structure designed to support confidence in conformity assessment activities, including medical laboratory services. At the regional level, the European Co-operation for Accreditation (EA) serves as the recognized accreditation infrastructure by coordinating national accreditation bodies (NABs) and ensuring the harmonized application of accreditation principles across participating economies [19,30]. EA operates the European Multilateral Agreement (EA-MLA), a formal arrangement under which accreditation decisions issued by signatory NABs are mutually recognized for defined technical fields, including medical laboratories accredited to the ISO 15189 standard [19].

It is worthwhile mentioning that participation in the EA-MLA is not limited to European Union Member States. Several non-EU countries are full signatories, meaning that EA-MLA represents a broader European and regional healthcare space rather than an exclusively EU regulatory mechanism. This distinction is relevant for healthcare systems that rely on cross-border recognition of laboratory results, patient mobility, and international public health cooperation.

At the international level, the EA-MLA aligns with the International Laboratory Accreditation Cooperation Mutual Recognition Arrangement (ILAC-MRA), which provides global recognition for accredited laboratory services within defined scopes, including medical laboratory services [18]. Together, these arrangements establish a pyramidal structure in which international and regional policies are implemented through national accreditation systems, while healthcare services are delivered by accredited laboratories at the operational level.

The methodological pathway was intentionally designed to reflect the governance structure of the EA MLA itself, rather than to rely on generic database searches. As a first step, the official EA MLA public registry was used to identify all NABs that are signatories to the EA Multilateral Agreement. For each signatory, the official website of the NAB was accessed directly via the link provided or referenced within the EA ecosystem.

Within each NAB website, document retrieval followed a targeted and bounded approach. Under EA rules, each NAB maintains a single authoritative regulatory document (e.g., regulation, policy, procedure, or guideline) governing the use of the accreditation symbol and/or reference to accreditation by accredited conformity assessment bodies. Consequently, for each country, only one governing document was eligible for inclusion. This structural constraint substantially reduced the risk of document misidentification and ensured conceptual consistency across jurisdictions.

Verification, according to which each retrieved document was current and in force, was based on institutional governance requirements rather than ad hoc assumptions. All EA MLA signatory NABs are required to comply with ISO/IEC 17011 [30], compliance with which NAB is assessed through periodic peer evaluation at the EA level, typically every four years. As part of this framework, NABs are explicitly required to make publicly available—and to keep updated—information on the use of the accreditation symbol and claims of accreditation.

In particular, ISO/IEC 17011:2017 [30], Clause 8.2.1.6, requires accreditation bodies to publish and update, without any request, information on the use of the accreditation symbol or other claims of accreditation. Consequently, for each country, only one governing document was eligible for inclusion. Accordingly, documents published on official NAB websites were treated as the current and operative versions, unless explicitly marked as superseded or archived. Document codes, revision numbers, and publication or applicability dates were recorded to preserve traceability.

Where available, official English or bilingual versions of NAB documents were used directly. In cases where multiple language versions were provided, the English or French version published by the NAB was preferred. No interpretative upgrading or harmonization of wording was performed. The Extraction focused strictly on explicit normative provisions (e.g., “*shall*,” “*must*,” “*may*”) governing the use of accreditation reference. Clause identifiers were retained for every extracted element in order to allow direct verification against the source document. The analysis avoided inferential interpretation and relied exclusively on express regulatory statements.

Document screening was performed at the NAB level. For each country, the governing document was screened for provisions addressing: (i) identification of accredited results on reports or certificates; (ii) conditions under which results may be issued without reference to accreditation; (iii) requirements for handling mixed accredited and non-accredited results; (iv) permitted uses of accreditation references beyond test reports.

The Extraction was performed by a single reviewer using a predefined analytical grid. To ensure accuracy and legal neutrality, a systematic verification step was applied, whereby each extracted statement was checked against the original clause reference to confirm fidelity of wording and preservation of normative strength. The purpose of this verification was to ensure traceability and to prevent interpretative distortion, rather than to measure inter-coder agreement.

A critical methodological criterion was version validity. Only documents explicitly identified as in force as of January 2026 were included in the analysis. This cut-off was selected to ensure comparability, as EA updated its core policy governing accreditation symbol use and reference to accreditation in June 2025 (EA-3/01, Revision 08), requiring implementation at the national level [19].

No interpretative upgrading or downgrading of normative language was performed. Mandatory, conditional, or optional requirements were reported as using the exact normative strength expressed in the source documents. This approach ensured that the analysis remained descriptive, traceable, and legally neutral, while allowing comparison of how accreditation-related communication is operationalized within healthcare documentation across the EA-MLA space.

This study is a descriptive, document-based mapping of accreditation communication rules and does not constitute a systematic review, nor does it evaluate compliance, effectiveness, or enforcement practices.

## 3. Results

The results are presented as a structured comparative overview of national rules governing how accreditation status is communicated in medical laboratory documentation across EA-MLA signatory countries. Rather than reporting findings on a country-by-country narrative basis, the analysis is synthesized in Table 1, which maps core rule dimensions derived from the governing national documents of each accreditation body. Table 1 summarizes how accredited results are identified, under which conditions non-accredited reporting is permitted, how mixed accredited and non-accredited results must be handled, and the extent to which accreditation references may be used beyond formal test reports. Clause-level references are provided for traceability and verification.

A further cross-cutting clarification emerging from the analysis concerns the formal distinction between the *accreditation body logo* and the *accreditation symbol*, a distinction that is frequently blurred in practice but is normatively strict within the EA framework. Under EA-3/01, the accreditation body logo is defined as a graphical identifier reserved exclusively for use by the national accreditation body itself and serves only to identify the authority of the NAB as an institution. By contrast, the accreditation symbol is a composite mark that incorporates the NAB identity together with a unique identifier of the accredited conformity assessment body (e.g., accreditation number or code) and, where applicable, the relevant accreditation field. Only this accreditation symbol—or an equivalent prescribed textual reference—may be used by accredited laboratories to signal that specific results fall within the accredited scope and benefit from EA-MLA recognition. Consequently, the regulatory provisions analyzed in this study uniformly govern the use of the accreditation symbol and textual references to accreditation, not the use of the NAB logo. This distinction is not semantic, but functional: the use of the logo by laboratories is explicitly prohibited, while the use of the accreditation symbol is tightly regulated to ensure traceability, to prevent misleading claims, and to preserve the legal integrity of accredited healthcare information.

**Figure 2:**
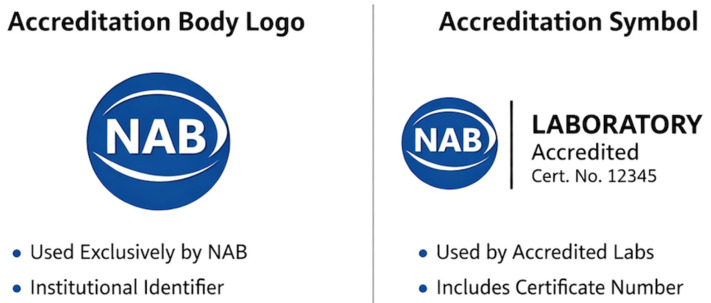
Illustrative distinction between the accreditation logo and the accreditation symbol.

Figure 2 illustrates the distinction between the accreditation logo (reserved for the accreditation body), the accreditation symbol (authorized for use by accredited laboratories), and their placement within medical laboratory reports:

Beyond the rule dimensions operationalized in Table 1, EA-3/01 Rev.08 establishes mandatory constraints governing the way in which accreditation may be communicated. Accreditation references must not imply responsibility, endorsement, or approval by the accreditation body for clinical results, medical decisions, products, or services; accreditation is strictly a statement of conformity assessment competence within a defined scope. In addition, EA requires immediate and complete withdrawal of all accreditation references—across reports, websites, and communication materials—whenever accreditation is suspended, reduced, expired, or withdrawn. These requirements constitute a mandatory governance layer that conditions the application of all table-level rules and explains the high degree of structural convergence observed across the EA-MLA, despite national granular differences.

Within this framework, strong convergence is also evident regarding restricted uses of accreditation references. The controlled use of websites and promotional or communication materials linked to accredited activities is permitted in the large majority of jurisdictions (approximately 38–40 of 42), subject to strict conditions on scope clarity and non-misleading presentation. By contrast, explicit prohibitions on the use of business cards appear in roughly 30–33 countries, while prohibitions on the use of products, labeling, or objects implying approval or endorsement are even more widespread (approximately 34–36 countries). Across all reviewed national rules, the immediate cessation of accreditation references following any suspension or withdrawal is uniformly required. Together, these patterns reflect a shared EA-level governance baseline, within which national accreditation bodies implement more detailed restrictions shaped by local, legal, institutional, and cultural contexts.

## 4. Discussion

Across the analyzed healthcare systems, the consequences associated with accreditation-related communication do not operate in a binary manner, but rather along a practical spectrum. In some contexts, the presence of accredited status functions primarily as an eligibility condition, determining whether laboratory results may be accepted for reimbursement or participation in regulated care pathways. In other systems, accreditation constitutes a formal requirement for service provision; that is, accredited status represents a prerequisite rather than a conditional attribute. In additional settings, accreditation-related references serve a more neutral administrative role, supporting transparency and information usability without directly triggering regulatory or financial consequences. This variation reflects how national rules operationalize accreditation communication within healthcare systems and is consistent with the coding framework applied in the present comparative analysis.

The national rules governing accreditation-related communication can also be understood as addressing distinct categories of communication risk within healthcare information systems. One recurrent risk concerns misinterpretation when accredited and non-accredited results are combined within the same report, which may lead users to incorrectly assume uniform status or recognition. A second category relates to the overclaiming or “*accreditation inflation*,” where references to accreditation could imply a broader scope, endorsement, or responsibility than formally granted. Additional rules target branding or promotional misuse, aiming to prevent accreditation symbols or references from being used in ways that resemble marketing claims rather than neutral information signals. Finally, several frameworks explicitly address ambiguity arising during suspension, reduction, or withdrawal of accreditation, and therefore require the immediate cessation or clear limitation of accreditation references in order to avoid misleading healthcare stakeholders. Together, these categories illustrate how accreditation communication rules function as preventive mechanisms within healthcare information governance rather than as evaluative or enforcement tools.

The communication controls observed across national frameworks are designed to mitigate these categories of risk primarily through clarity, restriction, and conditional use of accreditation references. Requirements for explicit visual or textual differentiation between accredited and non-accredited results directly address the risk of misinterpretation in mixed-scope reports. Limitations on the contexts in which accreditation symbols or wording may appear—particularly prohibitions on the use in branding, advertising, or product-related materials—aim to prevent overclaiming and promotional misuse of accredited status. Provisions governing suspension, reduction, or withdrawal typically require the immediate cessation or explicit qualification of accreditation references, thereby reducing ambiguity regarding the current status of reported results.

At the same time, the analysis indicates that communication controls cannot fully eliminate all potential ambiguity, particularly in complex service arrangements, cross-border information flows, or contractual exceptions explicitly allowed by national rules. These residual areas reflect contextual and organizational variability rather than regulatory absence, underscoring that accreditation-related communication operates as a risk-mitigation mechanism within healthcare information governance rather than as a guarantee against all forms of misinterpretation.

The findings also have implications for the international acceptance of healthcare laboratory information, particularly in distinguishing administrative from clinical use of test reports. Within multilateral recognition frameworks, accreditation-related communication primarily facilitates the administrative acceptance of laboratory results across borders, supporting regulatory recognition, reimbursement handling, and formal usability within healthcare systems. However, the international recognition of accredited status does not imply uniform clinical interpretation or direct transferability of clinical decision-making. The clinical use of laboratory information remains dependent on the local clinical guidelines, healthcare practices, and patient-specific contexts. Therefore, in cross-border settings, accreditation communication functions mainly as an information governance mechanism that enables administrative trust and system interoperability, while clinical responsibility and interpretation continue to reside at the national or local level.

This comparative review shows that, across the EA-MLA space and adjacent jurisdictions, rules on the way in which accreditation is communicated function as a practical layer of healthcare information governance. The technical act of producing a laboratory result is not the only determinant of how that result is used; the reporting conventions that indicate (or do not indicate) accredited status shape how results are interpreted, accepted, and processed within clinical pathways and administrative workflows.

A consistent cross-country pattern is that accreditation is operationalized at the report level, most commonly via an accreditation symbol and/or standardized wording. This has direct consequences for healthcare management because report-level signals are what downstream users actually see: clinicians triaging decisions, managers tracking service performance, and payers applying reimbursement rules. Therefore, accreditation communication becomes a “transactional attribute” of laboratory information, affecting routine decision points rather than remaining a background quality concept.

A second convergent element is the mandatory differentiation when accredited and non-accredited content appears within the same document. From a patient-safety and operational perspective, this requirement reduces ambiguity in mixed-service environments and supports consistent handling of results in settings such as referrals, second opinions, outsourced testing, and public health programs. Importantly, several frameworks also define conditions under which a laboratory may issue results without any accreditation reference, but these clauses typically require explicit disclosure that the report is not issued as accredited. This supports transparency and avoids implicit upgrading of report status.

The analysis also highlights the health-financing relevance of these communication rules. In some systems, accredited status is closely linked to eligibility for reimbursement or participation in structured care pathways; therefore, the way in which accredited status is displayed (or excluded) can influence access to payment streams and, indirectly, patient access to services. Conversely, stricter regimes treat accreditation as a baseline condition for service provision by making accreditation-related communication part of routine compliance and service continuity.

Beyond laboratory test results, national documents regulate the appearance of accreditation references in channels increasingly used for patient and provider information—websites, brochures, correspondence, and contractual materials. The recurring policy logic is not promotional; it is risk control against misleading representations that could distort user understanding of service status, scope, or responsibility.

Finally, while national rules reflect a shared regional policy foundation, they differ in prescriptiveness and in the granularity of allowed “*use cases*”. These differences are unlikely to indicate divergent analytical quality, but they may affect cross-border intelligibility and administrative alignment. For healthcare leaders, the key implication is that standardized, non-misleading accreditation communication supports safer interpretation, more predictable reimbursement handling, and clearer accountability, which are core objectives in sustainable and efficient healthcare delivery.

From a governance perspective, the analysis suggests that clarity in accreditation-related communication benefits from consistent structuring of key informational elements across jurisdictions. Without proposing harmonized rules or policy changes, the findings indicate that certain minimum informational components—such as explicit identification of accredited scope, clear differentiation between accredited and non-accredited results, and unambiguous statements regarding accreditation status during suspension or withdrawal—are recurrent across national frameworks.

At the same time, the specific wording used to express these elements varies across jurisdictions, therefore, reflecting differences in national legal traditions, administrative culture, and language. English-language versions made available on institutional websites are typically provided to support accessibility and international understanding, while the authoritative legal or procedural formulations remain those adopted within national legal and administrative frameworks. Despite these textual differences, functional alignment is maintained through adherence to EA-level requirements, which define common structural principles while allowing national accreditation bodies to preserve legally and culturally appropriate formulations.

In order to support transparent communication of accredited status, the analysis highlights several recurrent principles reflected across national frameworks. These include: (i) clear identification of the accredited scope in order to avoid ambiguity regarding which of the activities or results are covered; (ii) explicit differentiation between accredited and non-accredited results when both appear within the same report or communication context; (iii) restraint in the use of accreditation references outside formal reporting contexts to prevent overclaiming or promotional misuse; and (iv) immediate clarification or cessation of accreditation references in cases of suspension, reduction or withdrawal. These elements are not discretionary choices of individual accreditation bodies, but reflect the implementation of common requirements articulated at the EA level and incorporated into national accreditation communication rules. They are presented here as a descriptive synthesis rather than as prescriptive guidance.

This review has several limitations that should be acknowledged. First, the analysis was intentionally restricted to accreditation-related communication rules as documented in official policies and procedures issued by national accreditation bodies. General healthcare financing statutes, reimbursement laws, or broader regulatory frameworks were not examined unless they were explicitly referenced within these accreditation documents. Second, the study did not assess real-world implementation, compliance, or effectiveness of the identified rules in practice. The analysis is descriptive and document-based, focusing on formal governance and communication requirements rather than on operational behavior or enforcement outcomes. Finally, while all documents were reviewed in their officially published versions, some materials were available only in national languages, and the interpretation relied on authorized translations or expert linguistic review, which may introduce minor nuances in wording.

## 5. Conclusions

This study provides a document-based analysis of accreditation-related communication rules as articulated in official policies issued by national accreditation bodies, focusing on governance and information management aspects within healthcare systems.

The analysis also identifies a small set of common, EA-derived elements that underpin transparent communication of accredited status across healthcare systems.

This study demonstrates that the communication of accreditation-related information in healthcare laboratory documentation operates as a functional component of healthcare management, financing, and policy implementation, rather than as a purely technical or symbolic feature. Across the EA-MLA space, national rules governing how accredited status is referenced in routine documentation directly shape the usability of healthcare information in clinical pathways, reimbursement mechanisms, and public health programs.

A key contribution of this work is its comprehensive scope. The analysis covers all EA-MLA signatories, encompassing all 27 EU Member States, eight associated countries, and six non-EU economies participating in the EA-MLA framework, using only officially valid documents in force as of January 2026. This provides, to our knowledge, the first fully updated, system-wide mapping of accreditation communication rules within the European accreditation infrastructure.

The findings confirm that accreditation is primarily encountered by healthcare stakeholders as a standardized communication signal incorporated in documentation, influencing eligibility for reimbursement, insurance acceptance, and regulated care routes. Variations in national rules reflect healthcare system organization and policy choices rather than differences in technical competence.

By framing accreditation communication as an element of healthcare information governance, this study offers a transparent reference base for healthcare managers, policymakers, and researchers, by supporting informed comparison, policy alignment, and future system-level improvement without compromising legal or institutional neutrality.

## Figures and Tables

**Table 1:** Accreditation symbol use and reporting rules across EA-MLA national accreditation bodies.

Country (NAB)	Governing Rule (Code, Year)	Identification of Accredited Results	Conditions for Non-Accredited Reporting	Handling of Mixed Results	Use Beyond Test Reports
Albania (DPA)	DA-PO-005, Rev.11 (2025) [31]	Mandatory symbol or text reference (Secs. 6–7)	Contractual omission with client notice (Sec. 7)	Mandatory differentiation with explicit marking (Secs. 6–7)	Permitted for accredited activities only (Secs. 6–7)
Algeria (ALGERAC)	PRO 19, Version 08 (2025) [32]	Mandatory reference for accredited scope (§5.1(b))	No routine omission foreseen (§§5.1–5.2)	Accredited activities clearly identified (§5.1(b); Annex 02)	Permitted for accredited activities only (§5.1(c)–(d); Annexes 01–02)
Azerbaijan (AzAK)	P014, Rev. 04 (2025) [33]	Mandatory symbol or textual reference (Art. 5.1)	No reference outside the accredited scope (Art. 5.3)	Accredited results clearly distinguished (Art. 5.4)	Permitted for accredited activities only (Art. 6.1–6.2)
Austria (AA)	Guideline L04 (2022) [34]	Symbol mandatory on accredited reports (Sec. 3.1.1)	Omission only by contractual exception (Sec. 3.1.5)	Mandatory visual and textual separation (Secs. 3.1.3–3.1.4)	Permitted on communications, scope-linked (Secs. 2.2–2.3)
Belgium (BELAC)	BELAC 2-001, Rev. 15 (2025) [35]	Symbol or wording sole accreditation identifier (Sec. 3.1.1; 3.4)	No reference if no accredited results (Sec. 3.1.1; 3.4.1)	Mandatory distinction accredited vs. non-accredited	Permitted on websites, promotional documents (Sec. 3.10)
Bulgaria (BAS)	BAS QR 5, Ver. 6 (2024) [36]	Symbol or text mandatory on accredited reports (Sec. 6; 7.3.1)	No reference if no accredited results (Sec. 6; 7.3.1)	Clear separation and mandatory disclaimers (Sec. 7.1.3; 7.3.2)	Permitted on websites, offers, correspondence (Sec. 7.2.1–7.2.2)
Croatia (HAA)	HAA-Pr-2/5 (2025) [37]	Symbol or text mandatory on reports (Art. 3.1; 4.1)	Omission allowed by documented agreement (Art. 3.1; 4.4)	Clear distinction of non-accredited results (Art. 3.1; 4.3–4.4)	Permitted with HAA approval only (Art. 2.2; 6)
Cyprus (CYS-CYSAB)	R 01, Issue 4.5 (2025) [38]	Symbol or text mandatory for accredited results (Annex A, Clauses 1.2; 4.4)	Omission allowed only by documented agreement (Annex A, Clause 1.2)	Clear marking of non-accredited results required (Annex A, Clauses 4.4(a–b); 4.6)	Permitted on websites and advertising (Annex A, Clauses 5.1–5.2)
Czechia ČIA	MPA 00-04-23 (2023) [39]	Symbol or textual reference mandatory (Clause 2.2.1)	Omission only by documented agreement (Clause 2.2.1; Note)	Mandatory clear differentiation required (Clauses 2.2.1; 2.2.6; 2.2.7)	Permitted on websites, letters, promotion (Clauses 2.2.2; 2.2.7; 3.1)
Denmark (DANAK)	AB 2 (2022) [40]	Symbol or text mandatory on reports (Secs. 3.1; 3.5; 2.5–2.6)	Omission allowed by a written client request (Sec. 3.6)	Mandatory separation of accredited activities (Secs. 3.9–3.10)	Permitted on letters, websites, advertising (Secs. 3.11–3.12)
Egypt (EGAC)	R4G, Issue 1.4 (2021) [41]	Symbol or text mandatory on reports (Clauses 4.1; 4.6)	Non-accredited results allowed with a disclaimer (Clauses 3.4; 4.6)	Mandatory visual separation and disclaimer (Clauses 4.3–4.6)	Permitted on stationery, websites, publicity (Clauses 1.5; 9.1; 9.3)
Estonia (EAK)	EAK J-09-03 (2025) [42]	Symbol or text mandatory on reports (Clauses 1.4; 4.1)	Omission allowed by law or contract (Clause 1.5)	Clear differentiation with mandatory disclaimer (Clauses 4.8; 4.13)	Permitted on websites, marketing materials (Clauses 3.9.3; 3.9.4)
Finland (FINAS)	FINAS V1/2025 (2025) [43]	Symbol or written reference mandatory (Sec. 5.2.1)	Omission allowed by the client agreement (Sec. 5.2.1)	Accredited results clearly distinguished (Sec. 5.2.1)	Permitted for accredited activities only (Secs. 5.3.1–5.3.3)
France (COFRAC)	GEN REF 11, 2026 [44]	Symbol or textual reference mandatory on reports (§7, §7.1–7.2)	Omission allowed only by documented agreement (§7)	Clear separation of accredited results required (§7.1.2-a, §7.1.2-d)	Permitted on websites and communications (§7.1.2-c, §7.2)
Greece (ESYD)	NAL/02/03, Rev.03 (2023) [45]	Accreditation symbol or text mandatory if any accredited test present (Clauses 1.2(b), 3.1)	Non-accredited results allowed only with explicit marking (Clause 3.1)	Mandatory separation; non-scope results clearly identified (Clauses 3.1, 3.3)	Permitted on websites, brochures, correspondence (Clauses 2.3, 4.1–4.2)
Germany (DAkkS)	SD-DAkkS-002, Rev.1 (2025) [46]	Symbol or text required on conformity reports (Sec. 3.1; EA-3/01 §5.2)	Omission allowed by individual contractual agreement (Sec. 3.1, para. 5)	Clear attribution; warnings for non-accredited outputs (Sec. 3.1; warning text specified)	Permitted on letterheads and websites (Secs. 3.1; 3.2.1)
Hungary (NAH)	NAR-08, Edition 12 (2025) [47]	Accreditation reference mandatory on accredited results (Sec. 3.1)	Omission allowed only by explicit agreement (Sec. 3.3.1)	Mandatory separation of accredited and non-accredited results (Sec. 3.3.2)	Permitted for accredited activities only (Secs. 3.1; 3.3.2; 3.1)
Iceland (ISAC)	ACR-0002, Issue 5 (2024) [48]	Symbol or text mandatory for accredited results (Clauses 6–7)	Omission allowed by written client agreement (Clause 6)	Clear distinction between accredited vs. non-accredited required (Clauses 13–15)	Permitted on websites, stationery, quotations (Clause 5)
Ireland (INAB)	INAB R1 (2025) [49]	Symbol or text mandatory for accredited results (Sec. 3.8)	Omission allowed only as non-accredited output (Sec. 3.8)	Accredited and non-accredited clearly distinguished (Secs. 3.8–3.9)	Use permitted for accredited activities only (Sec. 4.1)
Israel (ISRAC)	Procedure 1-455001, Version 21 (2025) [50]	Symbol or combined mark identifies accreditation (Clauses 5.3, 5.4; 7.3.1–7.3.2)	Reporting allowed without a symbol, no conformity claims (Clause 7.1.6; 7.7)	Mandatory visual distinction; asterisk and disclaimer (Clauses 7.3.3; 7.3.4; 7.3.6)	Permitted on websites, letters, promotions (Clauses 3.5; 7.10.6; 7.11)
Italy (ACCREDIA)	RG-09, Rev.12 (2024) [51]	Symbol or text mandatory for accredited results (RG-09, §6.4.8; §6.5.2.2)	Omission allowed by documented client agreement (RG-09, §6.5.2.16)	Mandatory marking of non-accredited results (RG-09, §6.5.2.3; §6.5.2.4)	Permitted on websites, promotion, vehicles (RG-09, §6.4.11; §6.4.12; §6.4.13)
Kosovo (DAK)	Admin. Instruction No. 02/2018 (2018) [52]	Symbol mandatory on accredited reports (Art. 2; Art. 5.1)	Non-accredited results allowed, clearly marked (Art. 5.2)	Asterisk marking and disclaimer required (Art. 5.2)	Not permitted (Art. 5.5)
Latvia (LATAK)	LATAK-D.011, Rev.10 (2025) [53]	Symbol or text mandatory for accredited results (Sec. 6.1; 6.2)	Omission allowed by explicit client agreement (Sec. 6.1)	Clear separation; accredited results identified (Sec. 6.3.3–6.3.4)	Permitted on websites, letters, and advertising (Sec. 7.1)
Lithuania (LA)	AD 5.5:2023 (2023) [54]	The accreditation symbol identifies accredited outputs (AD 5.5, §1.1; §4.2)	No accreditation claims without accredited scope (AD 5.5, §4.2.4)	No misleading combination of symbols ( AD 5.5, §4.2.4; §4.2.6)	Permitted on official communication materials (AD 5.5, §4.2.8–§4.2.9)
Luxembourg (OLAS)	A003, Version 19 (2025) [55]	Symbol or text required on accredited reports (Clauses 4.1, 4.3)	Non-accredited reports prohibited from reference (Clause 4.8)	Accredited and non-accredited results clearly distinguished (Clause 4.3)	Permitted on websites, letterheads, advertising (Clause 4.6)
Malta (NABMALTA)	RAB02, Rev.9 (2021) [56]	Symbol or text required on accredited reports (Cl. 3.5; 4.1; 4.5)	Omission allowed only by documented agreement (Cl. 3.5; 3.7)	Mandatory clear disclaimer for non-accredited results (Cl. 4.4; 3.10)	Permitted on websites and publicity, controlled (Cl. 6.1–6.4)
Montenegro (ATCG)	PA.02-1—Rules on accreditation marks (2023) [57]	Symbol or textual reference mandatory on accredited results (Art. 4.2.3.1)	No symbol allowed outside accredited scope (Art. 4.2.3.2)	Mandatory clear marking of non-accredited results (Art. 4.2.3.2)	Permitted on promotional materials for accredited activities (Art. 4.2.3.1; 4.2.3.2)
Norway (NA)	D00067 12.00, 2026 [58]	Accreditation symbol or textual reference mandatory on reports (Secs. 6.8; 5.1)	Reporting without reference allowed only by agreement (Secs. 6.1; 6.8)	Accredited and non-accredited results clearly distinguished (Secs. 6.4; 6.8; 6.10)	Permitted on websites and promotional materials (Secs. 6.5; 6.6)
Poland (PCA)	PCA DA-02, 2025 [59]	Mandatory symbol or textual reference (§1; §2.3; §6.1)	Exception by client request, PCA approval (§1; §5.1; Annex 2 B1)	Clear differentiation; disclaimers required (§4.7; Annex 1 A1–A3)	Permitted for accredited activities only (§2.3; §4.1–4.3)
Portugal (IPAC)	DRC002—(2022) [60]	Symbol mandatory on accredited result documents (Sec. 5.3.1)	No symbol permitted outside accredited scope (Sec. 5.3.1)	Mandatory marking of non-accredited results (Sec. 5.3.1)	Permitted for accredited activities promotion (Sec. 5.4.1–5.4.2)
Moldova (MOLDAC)	PM, Ed. 19 (2024) [61]	Symbol or textual reference identifies accredited results (P-08, §2; §2.1)	No accreditation reference for non-accredited outputs (P-08, §1; §2)	Clear distinction between accredited and non-accredited results (P-08, §2; §2.1)	Symbol permitted for accredited activities only (P-08, §1; §2)
North Macedonia (IARNM)	Reg. R 05, Ed. 14 (2024) [62]	Accreditation mark or reference mandatory (Art. 10; Art. 14)	Omission prohibited for accredited results (Art. 10; Art. 23)	Non-accredited results clearly marked (Art. 14; Art. 15; Art. 23)	Permitted for accredited activities only (Art. 20; Art. 22)
Romania (RENAR)	RE-02, 2025 [63]	Symbol or textual reference mandatory (Art. 6.2.1.2–6.2.1.4; 13.1)	Allowed only without a symbol; not recognized (Art. 6.2.1.16; 13.1)	Mandatory marking and disclaimer required (Art. 6.2.1.5; 6.2.1.9; 6.2.1.11)	Textual reference allowed; symbol prohibited (Art. 7.1.2; 9.2)
Serbia (ATS)	ATS-PA04 Rules, 2025 [64]	Symbol or textual reference mandatory (Clauses 5.6; 5.6.1)	Allowed only by documented agreement (Clause 5.6)	Mandatory clear marking of non-accredited (Clause 5.6)	Permitted for accredited activities only (Clauses 5.1; 5.7)
Slovakia (SNAS)	MSA-02, Ed. 8 (2024) [65]	Symbol or textual reference mandatory (§6.1; §7.1)	No reference if no accredited results. (§7.3.1)	Accredited/non-accredited “clearly distinguishable”. (§7.1; §7.3.2.1; §7.2.3)	Allowed if “at least partly” accredited. (§7.2.2; §7.2.3)
Slovenia (SA)	S05, Issue 16, 2021 [66]	Accreditation symbol or prescribed statement mandatory on accredited reports (Clauses 4.1; 4.2)	Omission allowed only by written agreement; results non-accredited (Clause 4.2)	Mandatory explicit marking of non-accredited results (Clauses 4.2; 4.3)	Permitted on websites and promotional materials for accredited activities (Clause 4.6)
Spain (ENAC)	CEA-ENAC-01, Rev. 29 (2025) [67]	Symbol or text mandatory on accredited reports (Clauses 6.1(a); 6.2(c))	Omission allowed only by documented client request (Clause 6.3(a)–(b))	Mandatory clear marking of non-accredited results (Clauses 6.3; 9.1(a))	Permitted on websites and promotional materials (Clauses 8(a); 5.1)
Sweden (SWEDAC)	STAFS 2020:1 (amended 2025) [68,69]	Symbol or text mandatory for accredited results (Sec. 12–13)	Omission allowed by explicit client agreement (Sec. 12)	Mandatory differentiation of accredited vs. non-accredited (Sec. 16)	Permitted on stationery, websites, vehicles (Sec. 15; Guidance §15.5)
Switzerland (SAS)	Document 739.ew, Rev.05 (2023) [70]	Symbol or text mandatory on reports (3.2a–b)	No accreditation reference if outside scope (3.2d)	Non-accredited services clearly identified (3.2c, 3.2f)	Advertising allowed with clear scope (3.3.1)
Turkiye (TÜRKAK)	R10.06, Rev.13 (2019) [71]	Symbol or written reference mandatory (Arts. 9.4, 12.1)	No symbol if no accredited results (Art. 9.4)	Mandatory marking and warning notes (Arts. 9.9, 9.18–9.19)	Permitted on promotion with limits (Arts. 7.1–7.6)
Netherlands (RvA)	ASR005-UK, Version 1.0 (2025) [72]	Accreditation mark or equivalent text mandatory (Arts. 15–16)	Omission only by documented client agreement (Art. 16.3)	Non-accredited results explicitly distinguished (Arts. 12; 16.2; 19)	Permitted on media with non-misleading scope ( Arts. 20–21)
Tunisia (TUNAC)	DO.G.05, Version 13 (2021) [73]	Symbol or text mandatory for accredited results (Clause 4; Clause 1)	Omission allowed by documented agreement only (Clause 1)	Non-accredited results explicitly marked and explained (Clause 5.2.5)	Permitted on websites and promotional materials (Clause 5.3.1)
Ukraine (NAAU)	IN-15.08.02, Rev.24 (2024) [74]	Symbol or accreditation statement mandatory (Cl. 4.1; 5.1)	Omission allowed by documented agreement only (Cl. 5.1)	Mandatory clear separation of accredited results (Cl. 4.1)	Permitted on websites and corporate materials (Cl. 4.5–4.6)
UK (UKAS)	Accreditation Logo and Symbols, Feb 2024 [75]	Symbol or prescribed text mandatory on reports (4.1.7; 5.1)	Accredited and non-accredited must be distinguished (4.1.7)	Clear separation of accredited activities is required (4.1.7; 4.1.9)	Permitted on publicity for accredited services (4.2.1)

## Data Availability

No new data were created or analyzed in this study.

## Abbreviations

The following abbreviations are used in this manuscript:

ACCREDIA	Italian Accreditation Body
ALGERAC	Algerian Accreditation Body
AzAK	Azerbaijan Accreditation Center
ATS	Accreditation Body of Serbia
ATCG	Accreditation Body of Montenegro
BAS	Bulgarian Accreditation Service
BELAC	Belgian Accreditation Body
CAB	Conformity Assessment Body
COFRAC	Comité Français d’Accréditation
CYS-CYSAB	Cyprus Accreditation Body
ČIA	Czech Accreditation Institute
DAkkS	German Accreditation Body
DAK	Kosovo Accreditation Directorate
DANAK	Danish Accreditation Fund
DPA	Directorate of Accreditation of Albania
EA	European Co-operation for Accreditation
EA-MLA	European Co-operation for Accreditation Multilateral Agreement
EAK	Estonian Accreditation Center
EGAC	Egyptian Accreditation Council
ENAC	Spanish National Accreditation Body
ESYD	Hellenic Accreditation System
EU	European Union
FINAS	Finnish Accreditation Service
HAA	Croatian Accreditation Agency
IAF	International Accreditation Forum
IARNM	Institute for Accreditation of the Republic of North Macedonia
ILAC	International Laboratory Accreditation Cooperation
ILAC-MRA	International Laboratory Accreditation Cooperation Mutual Recognition Arrangement
INAB	Irish National Accreditation Board
ISO	International Organization for Standardization
ISRAC	Israel Laboratory Accreditation Authority
ISAC	Icelandic Accreditation Body
LATAK	Latvian National Accreditation Bureau
LA	Lithuanian Accreditation Bureau
MOLDAC	National Accreditation Center of the Republic of Moldova
NAAU	National Accreditation Agency of Ukraine
NAH	National Accreditation Authority of Hungary
NAB	National Accreditation Body
OLAS	Luxembourg Accreditation and Surveillance Office
PCA	Polish Center for Accreditation
RENAR	Romanian Accreditation Association
RvA	Dutch Accreditation Council
SA	Slovenian Accreditation
SAS	Swiss Accreditation Service
SNAS	Slovak National Accreditation Service
SWEDAC	Swedish Board for Accreditation and Conformity Assessment
TUNAC	Tunisian Accreditation Council
TÜRKAK	Turkish Accreditation Agency
UKAS	United Kingdom Accreditation Service
WHO	World Health Organization
